# P-1319. Antimicrobial Activity of Aztreonam-Avibactam and Molecular Characterization of Enterobacterales Resistant to Ceftazidime-Avibactam and/or Meropenem-Vaborbactam from United States Medical Centers (2016-2024)

**DOI:** 10.1093/ofid/ofaf695.1507

**Published:** 2026-01-11

**Authors:** Helio SaderJohn Kimbrough, Zachary Kockler, Dmitri Debabov, Rodrigo E Mendes, Mariana Castanheira

**Affiliations:** Element Iowa City (JMI Laboratories), North Liberty, Iowa; Element Iowa City (JMI Laboratories), North Liberty, Iowa; Abbvie, Irvine, California; Element Iowa City (JMI Laboratories), North Liberty, Iowa; Element, North Liberty, IA

## Abstract

**Background:**

Aztreonam-avibactam (ATM-AVI) was approved by the United States (US) Food and Drug Administration (FDA) in February 2025. ATM-AVI has shown potent activity against multidrug-resistant (MDR) Enterobacterales, including metallo-β-lactamase (MBL) producers. We evaluated activity of ATM-AVI and comparators against isolates nonsusceptible (NS) to ceftazidime-avibactam (CAZ-AVI) and/or meropenem-vaborbactam (MEM-VAB) and against MBL producers.

**Table 1.**
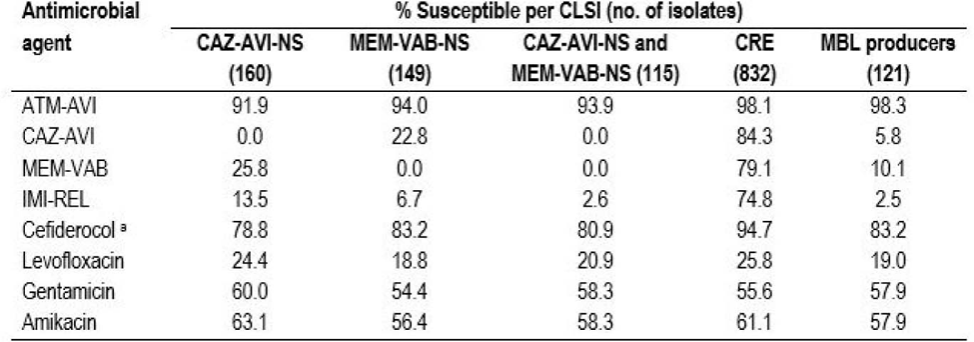
Antimicrobial susceptibility of resistant subsets Abbreviations: CAZ-AVI, ceftazidime-avibactam; NS, nonsusceptible; CRE, carbapenem-resistant Enterobacterales; MBL, metallo-β-lactamase; MEM-VAB, meropenem-vaborbactam; ATM-AVI, aztreonam-avibactam; IMI-REL, imipenem-relebactam.

**Figure:**
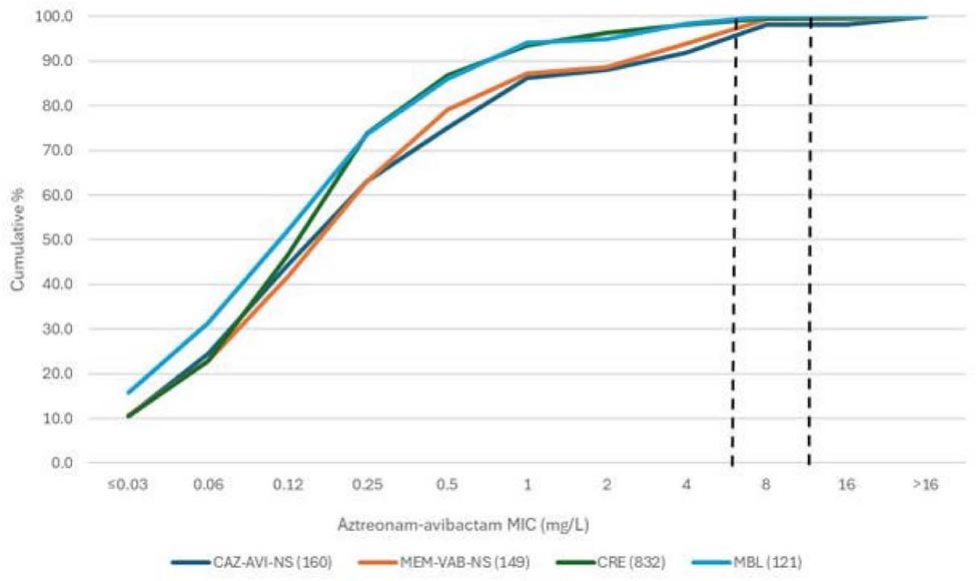
Finlandogram showing the activity of aztreonam-avibactam against selected resistant subsets.

**Methods:**

80,927 Enterobacterales isolates were consecutively collected (1/patient) in 2016–2024 from 103 US medical centers. Among these isolates, 194 (0.24%) were NS to CAZ-AVI or MEM-VAB, 115 (0.14%) were NS to both CAZ-AVI and MEM-VAB, and 832 isolates were carbapenem-resistant (CRE). Isolates were susceptibility tested by broth microdilution and screened for carbapenemase (CBase) genes by whole genome sequencing. Cefiderocol was tested in iron-depleted media. US FDA breakpoints were applied for ATM-AVI.

**Figure:**
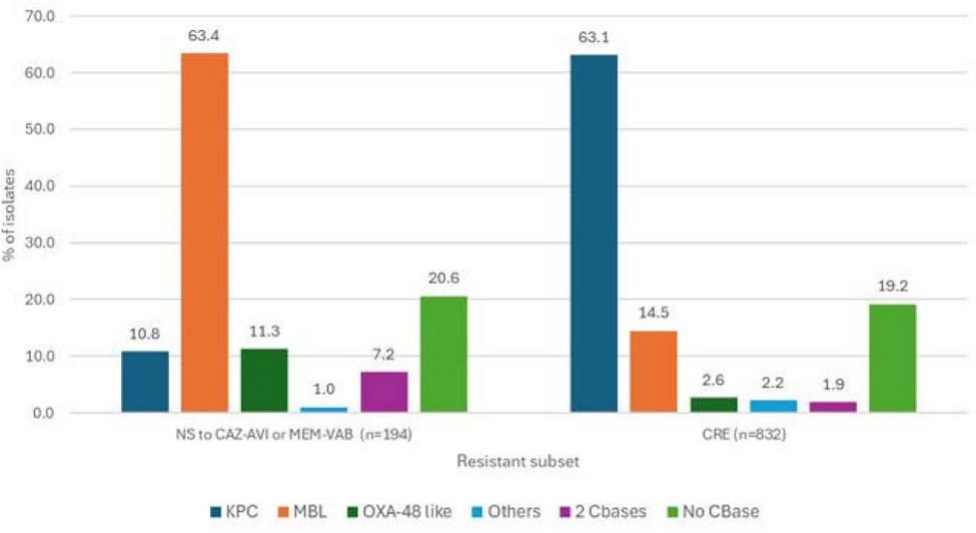
Distribution of carbapenemase (CBase) types Abbreviations: NS, nonsusceptible; CAZ-AVI, ceftazidime-avibactam; MEM-VAB, meropenem-vaborbactam; CRE, carbapenem-resistant Enterobacterales.

**Results:**

ATM-AVI was active (MIC ≤4 mg/L) against 91.9% of CAZ-AVI-NS, 94.0% of MEM-VAB-NS, 98.1% of CRE and 98.3% of MBL-producing isolates (Table 1 and Figure 1). Cefiderocol retained activity against 78.8% of CAZ-AVI-NS, 83.2% of MEM-VAB-NS, 94.7% of CRE and 83.2% of MBL-producing isolates. Amikacin was the most active compound among non-β-lactams, it inhibited 63.1% of CAZ-AVI-NS and 61.1% of CRE isolates at CLSI susceptible breakpoint. A CBase was identified in 154 (79.4%) of isolates NS to CAZ-AVI or MEM-VAB and 673 (80.9%) of CREs. NDM (n=110; 56.7% of isolates) was the most common CBase type among isolates NS to CAZ-AVI or MEM-VAB, whereas KPC (n=525; 63.1% of CREs) and NDM (n=109; 13.1%) were the most common CBase types among CREs (Figure 2). Notably, a MBL was identified in 34.2% (69/202) of CREs collected in 2023-2024.

**Conclusion:**

ATM-AVI demonstrated potent activity against isolates NS to CAZ-AVI and/or MEM-VAB as well as against CRE isolates, including MBL producers. The activities of other β-lactamase inhibitor combinations and cefiderocol were compromised by the increasing occurrence of MBL producers in US medical centers.

**Disclosures:**

Helio Sader, United States Food and Drug Administration: FDA Contract Number: 75F40123C00140 Rodrigo E. Mendes, PhD, GSK: Grant/Research Support|Shionogi & Co., Ltd.: Grant/Research Support|United States Food and Drug Administration: FDA Contract Number: 75F40123C00140 Mariana Castanheira, PhD, Melinta Therapeutics: Advisor/Consultant|Melinta Therapeutics: Grant/Research Support

